# Early Cardiotoxicity in Pediatric Patients with Acute Lymphoblastic Leukemia and Hodgkin Lymphoma: Incidence, Risk Factors, and Clinical Implications

**DOI:** 10.3390/children13101287

**Published:** 2026-09-22

**Authors:** Ronja Meyer, Leonie Dewein, Tobias Baumann, Katharina Rais, Daniel Steinbach, Michael Kaestner, Christian Apitz

**Affiliations:** 1 Section of Pediatric Cardiology, Hospital for Children and Adolescent Medicine, Ulm University Hospital, D-89075 Ulm, Germany; 2 Pediatric Hematology and Oncology, Hospital for Children and Adolescent Medicine, Ulm University Hospital, D-89075 Ulm, Germany

**Keywords:** pediatric oncology, cardiotoxicity, cardiooncology, acute lymphoblastic leukemia, Hodgkin lymphoma, cardiovascular complications

## Abstract

**Highlights:**

**What are the main findings?**

- Cardiovascular adverse events were detected in 69% of children with ALL and 68% of those with Hodgkin lymphoma, although most abnormalities were subclinical or transient.
- Clinically relevant complications included arterial hypertension, thromboembolic events, and rare treatment-requiring heart failure.

**What is the implication of the main finding?**

- Cardiovascular surveillance during and after pediatric cancer therapy is important to identify persistent or clinically relevant complications early.
- Prospective studies with standardized toxicity grading and detailed treatment- and patient-level risk assessment are needed to better characterize cardiovascular risk.

**Abstract:**

Background: Cardiotoxicity is a major adverse effect of antineoplastic therapy in pediatric oncology, potentially leading to long-term morbidity. This study aimed to evaluate the incidence, spectrum, and risk factors of early cardiovascular (CV) complications in children treated for acute lymphoblastic leukemia (ALL) and Hodgkin lymphoma (HL). Methods: In this retrospective cohort study, 109 patients diagnosed with ALL or HL between 2013 and 2019 were analyzed. All patients were under 18 years at diagnosis. CV assessment included electrocardiography, echocardiography, blood pressure and Holter monitoring, and additional imaging where available. Early cardiotoxicity was defined as CV events occurring during or shortly after therapy, including echocardiographic abnormalities, arrhythmias, arterial hypertension, and other CV complications. Descriptive and exploratory statistical analyses were performed to identify potential risk factors. Results: Early CV events occurred in 69% of ALL patients and 68% of HL patients. Multiple CV events were observed in 32% of ALL and 36% of HL patients. Most cardiotoxic effects were subclinical or transient; clinically significant CV complications were rarely detected. These comprised treatment-requiring heart failure, arterial hypertension, and thromboembolic events. Persistent CV abnormalities were also observed, including subclinical cardiac dysfunction, arterial hypertension, and cardiac arrhythmias. The majority of CV complications occurred within the first 500 days after therapy initiation. Increased cardiotoxicity was associated with older age, higher treatment risk groups, and disease relapse, while no significant sex differences were identified. Conclusions: CV abnormalities were frequent in children and adolescents receiving treatment for ALL or HL, although most were subclinical or transient. Clinically significant CV complications were uncommon. These findings support systematic CV surveillance during and after pediatric cancer therapy, with particular attention to persistent abnormalities and potentially treatable complications.

## 1. Introduction

Survival rates in pediatric oncology have improved substantially over recent decades, with 5-year survival exceeding 80% in many high-income countries [1,2,3]. As a consequence, treatment-related toxicities have emerged as a major determinant of long-term morbidity and mortality. Among these, cardiovascular complications are of particular clinical importance, as they represent a leading cause of non-cancer-related death in survivors of childhood cancer [2].

Cardiotoxicity is most commonly associated with anthracycline chemotherapy, which remains a central component of treatment protocols for acute lymphoblastic leukemia (ALL) and Hodgkin lymphoma (HL) [4,5]. The risk of cardiac injury is strongly influenced by cumulative drug exposure, with anthracyclines demonstrating a well-established dose-dependent toxicity profile [6,7]. In addition, newer cancer therapies have expanded the spectrum of cardiovascular complications, highlighting the need for broader cardio-oncological surveillance [8].

The pathophysiology of cancer therapy–related cardiotoxicity is complex and multifactorial. Anthracyclines induce myocardial injury through mechanisms including the generation of reactive oxygen species, mitochondrial dysfunction, and topoisomerase IIβ–mediated DNA damage, ultimately leading to cardiomyocyte apoptosis and impaired cardiac function [7,9]. These processes may begin shortly after exposure and initiate a cascade of structural and functional changes that progress over time.

Traditionally, cardiotoxicity in pediatric oncology has been considered a late complication, often manifesting years or decades after completion of therapy. However, emerging evidence suggests that cardiovascular injury frequently begins during treatment or shortly thereafter. Early-onset cardiotoxicity, including subclinical cardiac dysfunction, has been increasingly recognized, although its true incidence remains difficult to quantify due to heterogeneous definitions and monitoring strategies [4]. Importantly, early cardiac alterations may represent the first stage of a continuum that ultimately leads to clinically overt cardiomyopathy [7].

Despite increasing awareness, significant gaps remain in the understanding of early cardiotoxicity in contemporary pediatric oncology. Most available data focus on long-term outcomes or highly selected cohorts, while real-world data on early cardiovascular complications remain limited. In addition, the impact of systematic multimodal cardiac monitoring on detection rates has not been fully elucidated.

A more detailed characterization of early cardiotoxicity is therefore essential. Early detection of subclinical cardiac dysfunction may enable timely intervention and prevention of irreversible myocardial damage. Furthermore, identification of high-risk patients could facilitate risk-adapted surveillance strategies.

The aim of this study was to evaluate the incidence, spectrum, and potential risk factors of early cardiotoxicity in pediatric patients with ALL and HL treated under contemporary clinical conditions.

## 2. Methods

This retrospective cohort study included pediatric patients younger than 18 years who were diagnosed with ALL or HL between 2013 and 2019 and treated in our University Hospital. The study was conducted in accordance with the Declaration of Helsinki, and the protocol was approved by the Ethics Committee of the University of Ulm (Protocol No. 102/20; date of approval 4 May 2020). The analysis was restricted to cardiotoxicities associated with antineoplastic therapy in patients with ALL and HL, as these disease groups were expected to provide a sufficient number of cases and were characterized by relatively standardized treatment regimens according to established study protocols, allowing for adequate comparability of the data.

Patients with ALL were treated according to contemporary pediatric ALL protocols, predominantly AIEOP-BFM ALL 2009 (60/84, 71.4%) and AIEOP-BFM ALL 2017 (20/84, 23.8%), with a small number of patients treated according to Interfant 06 or the EsPhALL protocol. Patients with HL were predominantly treated according to the EuroNet-PHL framework: 18/25 patients (72.0%) according to EuroNet-PHL-C2, 5/25 (20.0%) according to EuroNet-PHL-C1, and 2/25 (8.0%) according to EuroNet-PHL-LP1. Treatment generally consisted of protocol-defined chemotherapy with response-adapted subsequent treatment; radiotherapy was administered to 7 patients with HL (28.0%).

Clinical data (demographics, clinical features, neoplastic diagnosis, therapeutic regimens, and cardiac evaluations) were extracted from medical records.

Cardiovascular monitoring was performed as part of routine clinical care assessments prior to, during, and after antineoplastic therapy and included electrocardiography, echocardiography, Holter monitoring, and ambulatory blood pressure measurements. In selected cases, cardiac magnetic resonance imaging was also performed. The frequency and type of investigations were based on clinical indications and institutional practice.

Early cardiotoxicity was defined as any cardiovascular abnormality detected during treatment or within the early follow-up period after therapy initiation. This included both symptomatic and asymptomatic findings. Newly occurring echocardiographic abnormalities included myocardial dysfunction, comprising systolic and diastolic functional impairment, myocardial hypertrophy, left ventricular dilatation, structural myocardial abnormalities, septal dyskinesia, aortic dilatation, dilatation of the vena cava and pulmonary arteries, right ventricular dysfunction, increased flow velocity in the pulmonary artery, and valvular dysfunction.

The electrocardiographic abnormalities identified in the analysis included bradyarrhythmias and tachyarrhythmias, QT interval prolongation, newly developed bundle branch block patterns, repolarization abnormalities, and ectopic electrical activity.

Other cardiovascular adverse effects associated with antineoplastic therapy included clinically manifest heart failure requiring treatment, myocarditis, pericarditis, arterial hypertension, thromboembolic events, newly developed pericardial effusions, and pulmonary hypertension.

Furthermore, the latency to the onset of cardiotoxic adverse effects, defined as the number of days from initiation of antineoplastic therapy to the first occurrence of cardiotoxicity, was recorded. For patients with disease relapse, the time to onset of a cardiotoxic adverse event was calculated as the interval from the initial diagnosis.

The clinical course of cardiovascular adverse effects was also assessed. For this purpose, cardiotoxicities were classified as transient events occurring during antineoplastic therapy, cardiovascular adverse effects persisting beyond completion of antineoplastic therapy, or late-onset toxicities developing after the end of chemotherapy.

The analysis distinguished subclinical abnormalities from clinically manifest cardiovascular events and specifically identified events requiring pharmacological treatment, intensive care, or persistent treatment beyond completion of oncological therapy.

For the analysis of risk factors for cardiotoxicity, the influence of age, gender, risk score, and presence of relapse was evaluated. Risk assessment was performed according to the AIEOP-BFM-ALL study protocols, stratifying into Low Risk (LR), Standard Risk (SR), Medium Risk (MR), and High Risk (HR). Risk assessment analysis was not performed for patients with HL owing to the anticipated small sample size within the respective risk subgroups, which precluded meaningful statistical evaluation.

### Statistical Analysis

Statistical analyses were primarily descriptive. The occurrence of cardiotoxic adverse effects was summarized using absolute and relative frequencies. Continuous variables were described using medians with minimum and maximum values, as well as means and standard deviations. Exploratory inferential analyses were performed using appropriate statistical tests, including Student’s *t*-test and, given the small sample sizes, the Wilcoxon test. All statistical tests were performed at a significance level of α = 0.05 and interpreted as exploratory and hypothesis-generating rather than confirmatory. No adjustment for multiple testing was applied. Statistical significance testing was restricted to subgroups with more than five observations. In addition, descriptive analyses using box plots were performed to evaluate the latency to the onset of cardiotoxic adverse effects in subgroups with more than 10 observations. Box plots present the median and the interquartile range (IQR), with the lower and upper boundaries of the box representing the 25th and 75th percentiles, respectively. Whiskers extend to the most extreme observations within 1.5 × IQR of the respective quartiles. Observations beyond 1.5 × IQR are displayed individually and may be considered outliers.

## 3. Results

A total of 109 patients (57.8% males) were included in the analysis, of whom 84 were diagnosed with ALL (55.9% males) and 25 with HL (64% males).

Among patients with ALL, the mean age at diagnosis was 7.3 years (median, 5.7 years; range, 0.08–17.9 years). Patients with HL had a mean age at diagnosis of 13.3 years (median, 14.3 years; range, 5.3–17.2 years).

The mean follow-up duration for the entire cohort was 5.6 years (median, 5.2 years; range, 2.6–9.4 years), providing sufficient longitudinal observation to evaluate both early and persistent cardiovascular sequelae.

Disease relapse occurred in 8 patients with ALL (9.5%) and 4 patients with HL (16.0%). One patient in each disease group subsequently developed a second malignancy.

Two patients had pre-existing cardiac disease prior to the diagnosis of their malignancy, both in the form of congenital heart disease. One patient had a complete atrioventricular septal defect, while the other had a perimembranous ventricular septal defect. Both patients had undergone corrective cardiac surgery during infancy.

At the time of the initial cardiological evaluation prior to antineoplastic treatment, but obviously in context with the underlying oncological disease, 14 patients presented with cardiac abnormalities. These included pericardial effusion in 12 patients (11 with ALL and 1 with HL) and arterial hypertension requiring antihypertensive medication in 2 patients.

Early cardiovascular complications during oncological treatment were frequently observed in both disease groups. In patients with ALL, 69% experienced at least one cardiovascular event, while a similar proportion of 68% was observed in patients with HL. Furthermore, multiple cardiovascular events occurred in approximately one third of patients, indicating a considerable burden of cardiac involvement during therapy (Table 1).

The spectrum of cardiovascular abnormalities was broad. (Figure 1, Figure 2 and Figure 3). Subclinical cardiac dysfunction, affecting either systolic or diastolic function, represented the most common finding. However, clinically manifest heart failure was rare. Two patients with ALL developed acute, treatment-requiring heart failure during follow-up. Both patients required intensive care treatment and subsequently recovered completely. In one patient, severe left ventricular systolic dysfunction occurred with a minimum ejection fraction of 33%; intensive care treatment resulted in recovery of left ventricular function to an ejection fraction of 75%. In the second patient, acute heart failure was associated with moderate left ventricular dysfunction and grade II mitral regurgitation; left ventricular function subsequently recovered, with an ejection fraction of 61%. In the HL cohort, one patient developed clinically manifest heart failure approximately 79 days after initiation of therapy. Left ventricular systolic function was mildly reduced, with a minimum ejection fraction of 45%, and the patient developed exertional dyspnea corresponding to NYHA functional class II. Treatment with bisoprolol and lisinopril was initiated. Although ventricular function subsequently improved, lisinopril was continued beyond completion of oncological treatment.

Arterial hypertension was documented in 26 patients, including 23/84 patients with ALL (27.4%) and 3/25 patients with HL (12.0%). Antihypertensive treatment was initiated in 23 of the 26 affected patients, whereas 3 patients were managed conservatively with observation. Frequently used antihypertensive agents included angiotensin-converting enzyme inhibitors and calcium-channel blockers; 10 patients required combination therapy. A treatment-related etiology was suspected in 14 cases, frequently in association with corticosteroid exposure, although other potential contributing factors such as renal involvement or acute renal impairment were also documented.

Twenty-three thromboembolic events were documented: 16 in patients with ALL (19.1%) and 7 in patients with HL (28.0%). Nineteen events were catheter-associated thromboses, predominantly involving the internal jugular, brachiocephalic, or superior vena cava regions. Three patients developed cerebral sinus vein thrombosis, and one patient developed bilateral popliteal vein thrombosis despite prophylactic anticoagulation. Clinically relevant thrombotic events were treated with low-molecular-weight heparin according to the clinical course.

Arrhythmias and electrocardiographic abnormalities were documented in 23 patients with ALL (27.4%) and 5 patients with HL (20.0%). Recurrent tachyarrhythmias and bradyarrhythmias were the most frequent findings. Because of the retrospective nature of the study, the contribution of central venous catheter position to individual arrhythmic findings could not be systematically assessed in all patients.

Although most cardiovascular abnormalities were transient, persistent abnormalities were documented in a subset of patients. In the ALL cohort, persistent subclinical cardiac abnormalities, arterial hypertension, and arrhythmias/electrocardiographic abnormalities were observed during follow-up. In the HL cohort, one patient had persistent clinically manifest heart failure requiring ongoing pharmacological treatment beyond completion of oncological therapy. These findings indicate that a relevant proportion of cardiovascular abnormalities may persist after completion of antineoplastic treatment despite the predominantly transient nature of the overall cardiovascular toxicity burden (Figure 4).

The temporal analysis demonstrated that the majority of cardiovascular events occurred early, predominantly within the first 500 days after initiation of therapy. This pattern was consistent across both diagnostic groups (Figure 5).

Exploratory analyses suggested that older age at diagnosis, higher treatment intensity, and disease relapse were associated with an increased risk of cardiotoxicity (Figure 6). In contrast, no significant association between sex and the occurrence of cardiovascular complications was identified.

## 4. Discussion

The present study demonstrates a high burden of early cardiotoxicity in pediatric patients treated for ALL and HL, with cardiovascular abnormalities detected in more than two thirds of patients. These findings support the hypothesis that cardiotoxic effects are not limited to long-term survivorship but frequently emerge during active treatment and the early post-therapy phase. This aligns with accumulating evidence from large cohort studies and systematic reviews indicating that early-onset cardiotoxicity represents a clinically relevant and underrecognized phase of treatment-related cardiac injury [4].

A central observation of this study is the predominance of subclinical cardiac alterations, including mild ventricular dysfunction and asymptomatic arrhythmias. This supports the established paradigm that anthracycline-induced cardiotoxicity follows a continuum from early subclinical myocardial injury to overt heart failure [10]. Mechanistic studies have demonstrated that anthracyclines induce cardiomyocyte damage through reactive oxygen species generation and topoisomerase IIβ–mediated DNA injury, ultimately leading to progressive myocardial dysfunction [8,11]. Importantly, subclinical dysfunction has been shown to precede clinically apparent cardiomyopathy and is associated with adverse long-term outcomes [12]. Our findings extend this concept by demonstrating that these early alterations are highly prevalent already during therapy, even in contemporary treatment protocols.

The temporal clustering of cardiovascular events within the first 500 days after treatment initiation further supports the hypothesis that myocardial injury occurs early and is tightly linked to treatment exposure. Historical data from large cooperative group studies have shown that a substantial proportion of clinically overt cardiotoxicity develops within the first year after anthracycline exposure [13]. This early phase likely represents the transition from acute cellular injury to chronic structural remodeling. The biological plausibility of this observation is reinforced by experimental and clinical data demonstrating rapid onset of mitochondrial dysfunction, oxidative stress, and cardiomyocyte apoptosis following anthracycline administration [11,14].

Although most abnormalities in our cohort were transient, the persistence of cardiovascular alterations in a subset of patients is of particular clinical importance. Cardiotoxicity in childhood cancer survivors is increasingly recognized as a progressive and potentially irreversible process, with cardiovascular disease representing a leading cause of non-cancer-related morbidity and mortality in this population [15]. Even mild or asymptomatic ventricular dysfunction has been associated with an increased risk of heart failure later in life [12]. These findings support the concept that early cardiotoxicity may represent the initial stage of a long-term disease trajectory, thereby emphasizing the importance of early detection and longitudinal follow-up.

The identification of risk factors in this study is consistent with the established literature. The association between higher treatment intensity and increased cardiotoxicity reflects the well-documented dose-dependent relationship between anthracycline exposure and cardiac risk [7,14]. International guideline efforts have further refined this concept by introducing dose equivalence models across different anthracycline agents, highlighting substantial variability in cardiotoxic potential [16]. The increased risk observed in patients with relapse likely reflects cumulative exposure to cardiotoxic therapies, a finding that has been consistently reported in survivorship cohorts [16]. The association with older age may indicate differences in treatment regimens or myocardial susceptibility, although data on age-dependent vulnerability remain inconclusive. In contrast, the absence of a sex effect in our cohort differs from earlier reports and may be related to limited statistical power or population-specific characteristics [17].

From a clinical perspective, these findings have important implications for the evolving field of pediatric cardio-oncology. Contemporary guidelines emphasize that cardiovascular toxicity in childhood cancer patients extends beyond ventricular dysfunction and includes arrhythmias, hypertension, and vascular disease, necessitating a comprehensive and multimodal surveillance approach [7]. The high prevalence of subclinical abnormalities observed in our study underscores the need for sensitive diagnostic strategies, including advanced echocardiographic techniques and ambulatory monitoring [18]. Furthermore, recent advances in risk prediction models and surveillance frameworks highlight the importance of individualized, risk-adapted follow-up strategies [3,15].

The results of this study also support a shift in focus from reactive to proactive cardioprotection. Early identification of patients at increased risk may allow for timely implementation of preventive strategies, including dose modification, cardioprotective agents, and optimization of cardiovascular risk factors. While such approaches have shown promise, their integration into routine pediatric oncology practice remains variable and warrants further investigation.

### Limitations

The retrospective design limits causal inference; for example, the dataset did not allow for a standardized reconstruction of central venous catheter position or systematic assessment of catheter-related mechanical irritation as a potential cause of every arrhythmic event. Furthermore, standardized thrombophilia testing and comprehensive pre-treatment coagulation assessments were not available for all patients; an underlying congenital or acquired predisposition cannot be excluded. The thromboembolic findings should therefore be interpreted as multifactorial events potentially related to malignancy, treatment, central venous access, and individual patient characteristics.

A further limitation is the lack of individually verified cumulative anthracycline doses in the retrospective electronic dataset. Although treatment protocols allow the expected anthracycline exposure to be estimated, individual dose modifications, treatment interruptions, or deviations from protocol-defined therapy could not be reliably reconstructed for all patients. Consequently, associations between individual cumulative anthracycline exposure and cardiovascular outcomes could not be analyzed with sufficient accuracy.

Cardiovascular adverse events were not prospectively documented according to CTCAE version 4, and the available clinical records did not permit reliable retrospective grading of every event. We therefore refrained from assigning CTCAE grades where the required clinical information was incomplete. Instead, clinically relevant events were characterized according to their clinical manifestations, treatment requirements, need for intensive care, and persistence beyond completion of oncological therapy. This approach may underestimate or overestimate the severity of individual events compared with prospectively collected CTCAE-based assessments.

Although risk-adapted treatment is an integral component of pediatric Hodgkin lymphoma therapy, the retrospective dataset did not contain sufficiently complete patient-level information to reliably reconstruct standardized HL risk-group assignments for all 25 patients. We therefore considered it inappropriate to retrospectively assign patients to specific risk groups based on incomplete individual data. As a clinically meaningful alternative, we have now reported the distribution of the treatment protocols used in this cohort, with the majority of patients treated according to the EuroNet-PHL-C2 protocol. The small size of the HL cohort further limits meaningful statistical comparisons between risk groups.

Variability in diagnostic assessments may have influenced detection rates, particularly for subclinical findings. In addition, the single-center setting may limit generalizability. However, the real-world nature of the data reflects current clinical practice and highlights existing gaps in standardized cardiovascular surveillance.

In conclusion, cardiovascular abnormalities were frequently detected during and after treatment of pediatric ALL and HL, but most were subclinical or transient. The relatively low number of severe clinical events should not obscure the potential relevance of persistent abnormalities, particularly heart failure and arterial hypertension, or of thromboembolic complications. Our findings support systematic cardiovascular surveillance throughout treatment and during follow-up, while also emphasizing the need for prospective studies.

## Figures and Tables

**Figure 1:**
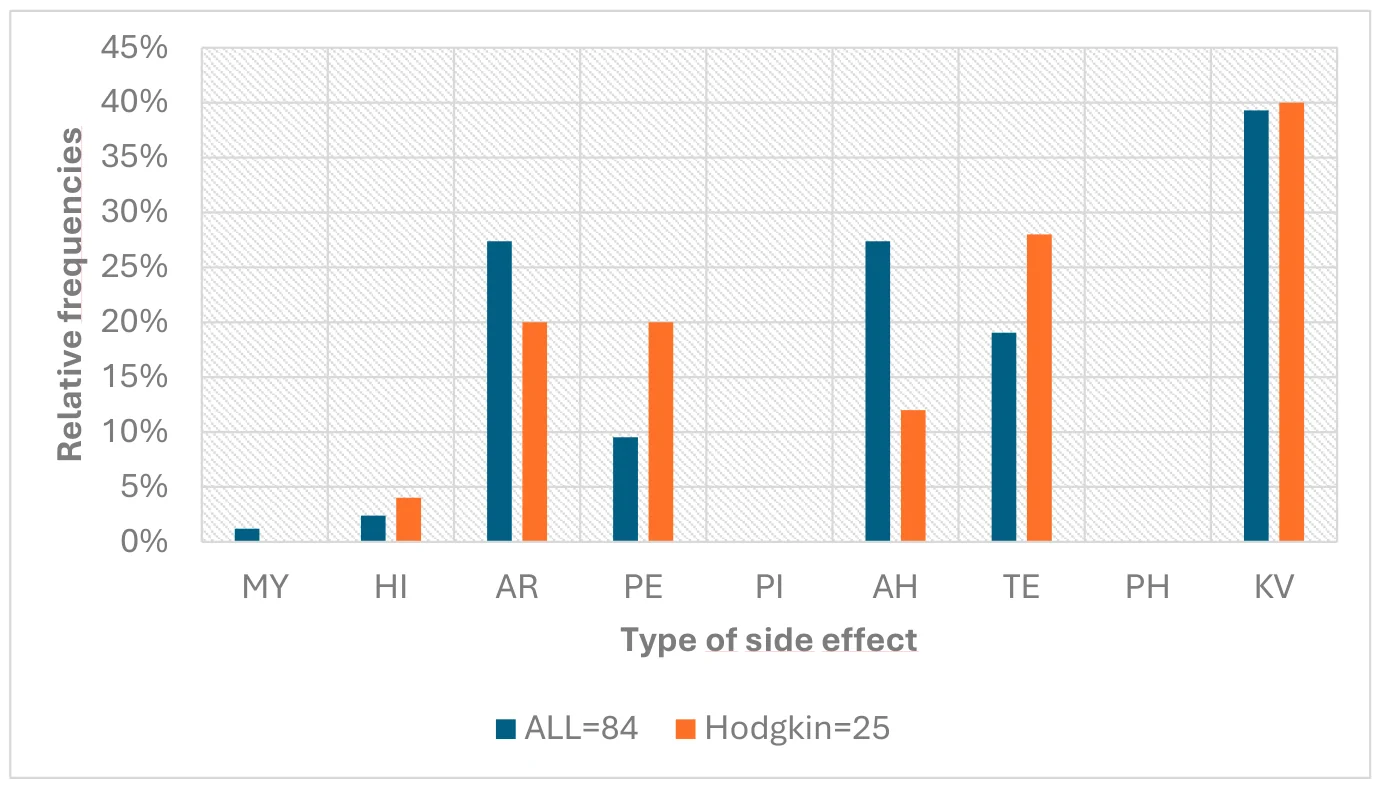
Type and frequency (relative frequencies) of cardiotoxic side effects of oncological therapies. Data related to the total collective of ALL and HL patients. Abbreviations: MY: myocarditis; HI: heart failure; AR: arrhythmias/ECG abnormalities; PE: pericardial effusion; PI: pericarditis; AH: arterial hypertension; TE: thromboembolism; PH: pulmonary hypertension; KV: subclinical cardiac alterations.

**Figure 2:**
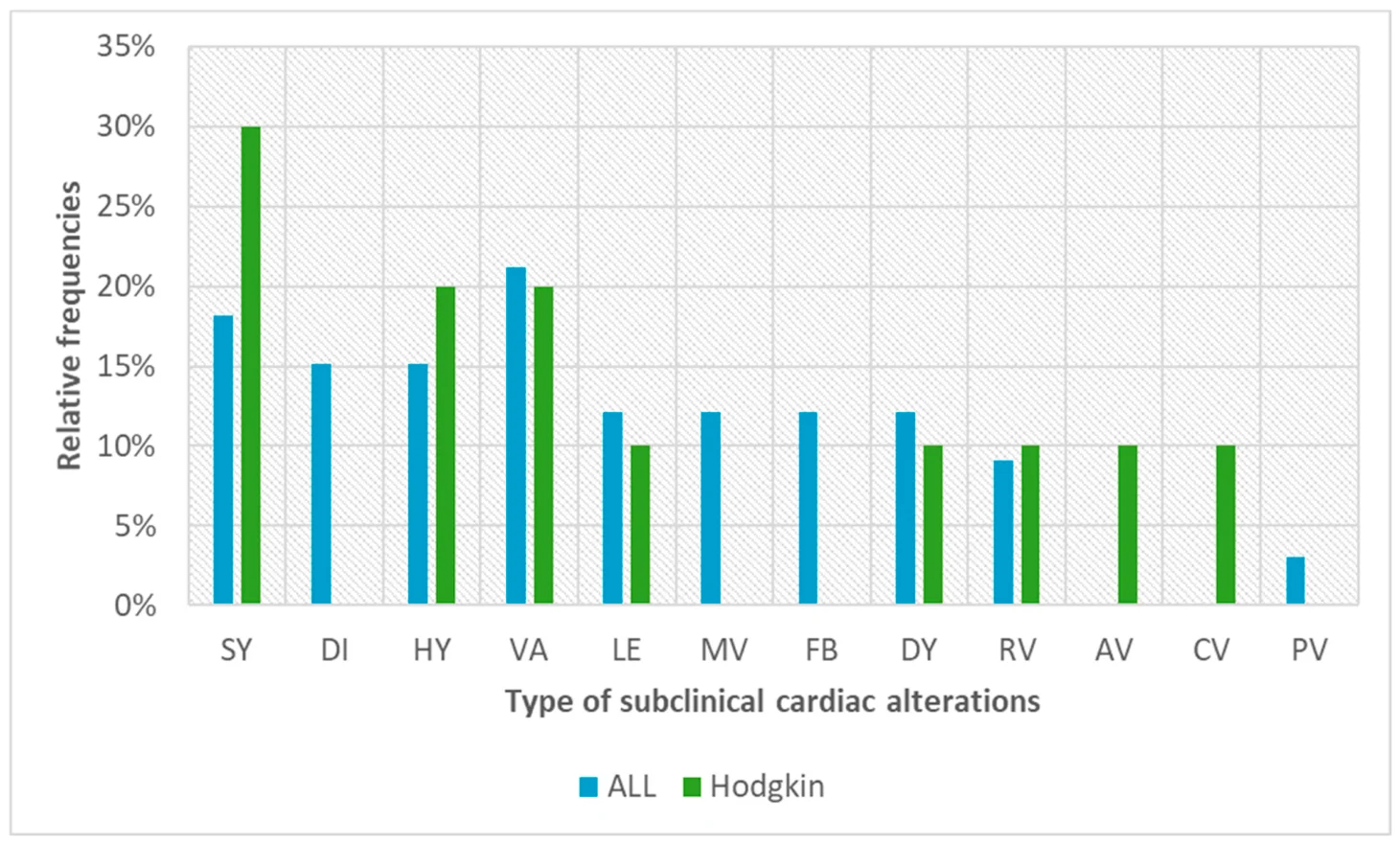
Type and frequency (relative frequencies) of subclinical cardiac alterations as a cardiotoxic side effect of oncological therapies in ALL and HL patients. Abbreviations: SY: systolic dysfunction; DI: diastolic dysfunction; HY: myocardial hypertrophy; VA: valvular dysfunction; LE: left ventricular dilatation; MV: structural myocardial abnormalities; FB: increased flow velocities; RV: right ventricular dysfunction; DY: septal dyskinesia; AV: aortic dilatation; CV: dilatation of the vena cava; PV: dilatation of the pulmonary artery.

**Figure 3:**
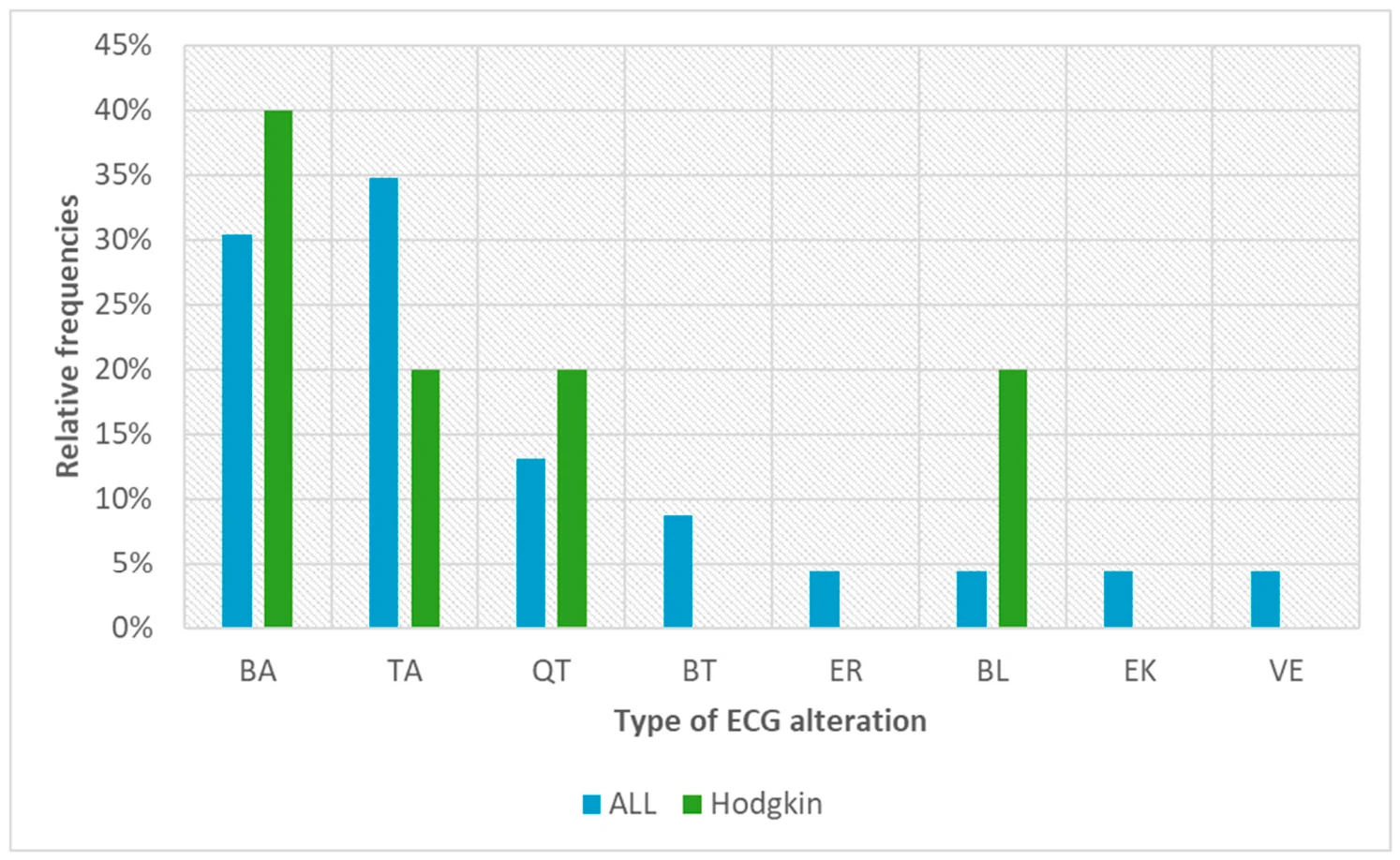
Type and frequency (relative frequencies) of ECG abnormalities and arrhythmias as a cardiotoxic side effect of oncological therapies in ALL and HL patients. Abbreviations: BA: bradyarrhythmias; TA: tachyarrhythmias; QT: QT interval prolongation; BL: newly developed bundle branch block patterns; ER: repolarization abnormalities; BT: tachy-brady arrhythmia; EK: ectopic electrical activity; VE: ventricular premature beats.

**Figure 4:**
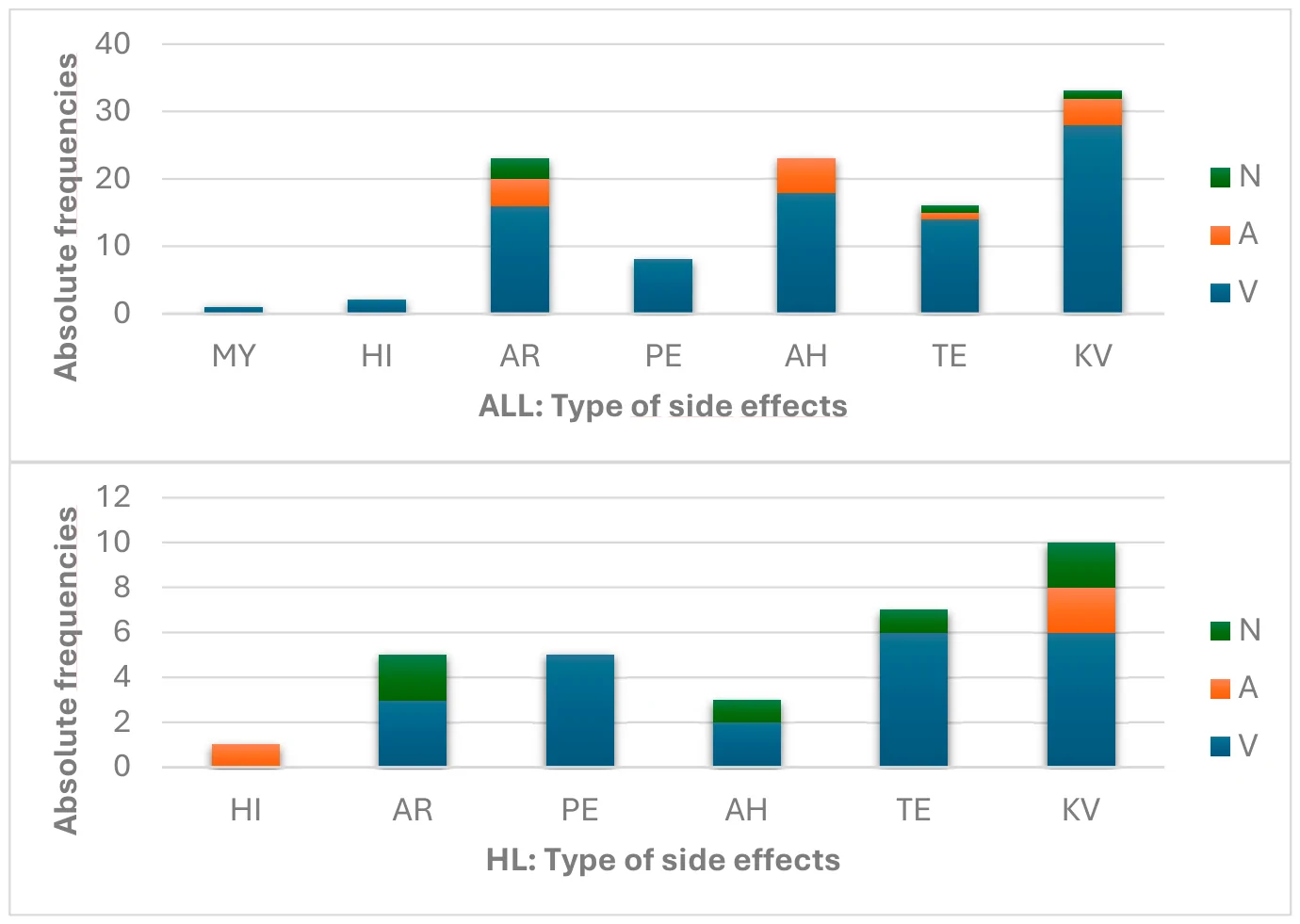
Course of the cardiotoxic side effects of oncological therapies in ALL and HL patients (absolute frequencies). Abbreviations: V: transient side effect; A: persistent side effect; N: side effect occurring after the end of therapy; MY: myocarditis; HI: heart failure; AR: arrhythmia/ECG abnormalities; PE: pericardial effusion; AH: arterial hypertension; TE: thromboembolism; KV: subclinical cardiac alterations.

**Figure 5:**
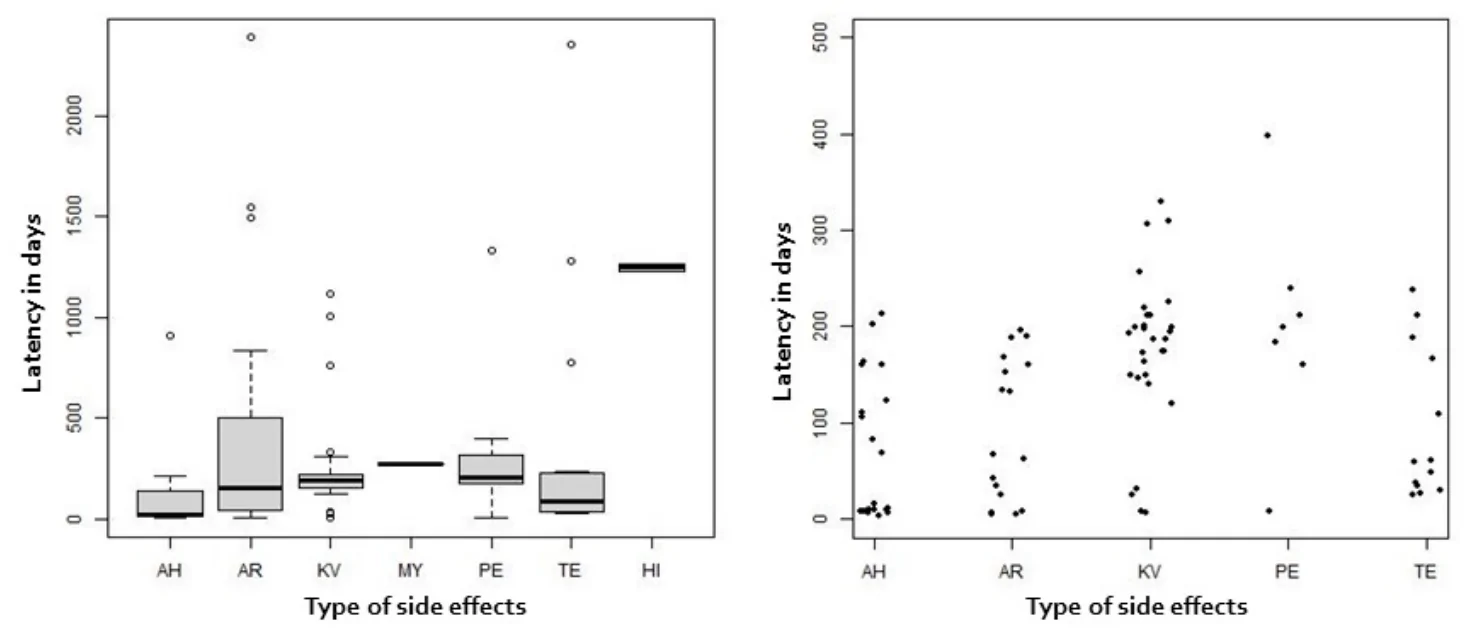
Latency to onset of cardiotoxic side effects of oncological therapies in ALL patients (latency stated in days). Abbreviations: MY: myocarditis; HI: heart failure; AR: arrhythmias/ECG abnormalities; PE: pericardial effusion; AH: arterial hypertension; TE: thromboembolism; KV: subclinical cardiac alterations.

**Figure 6:**
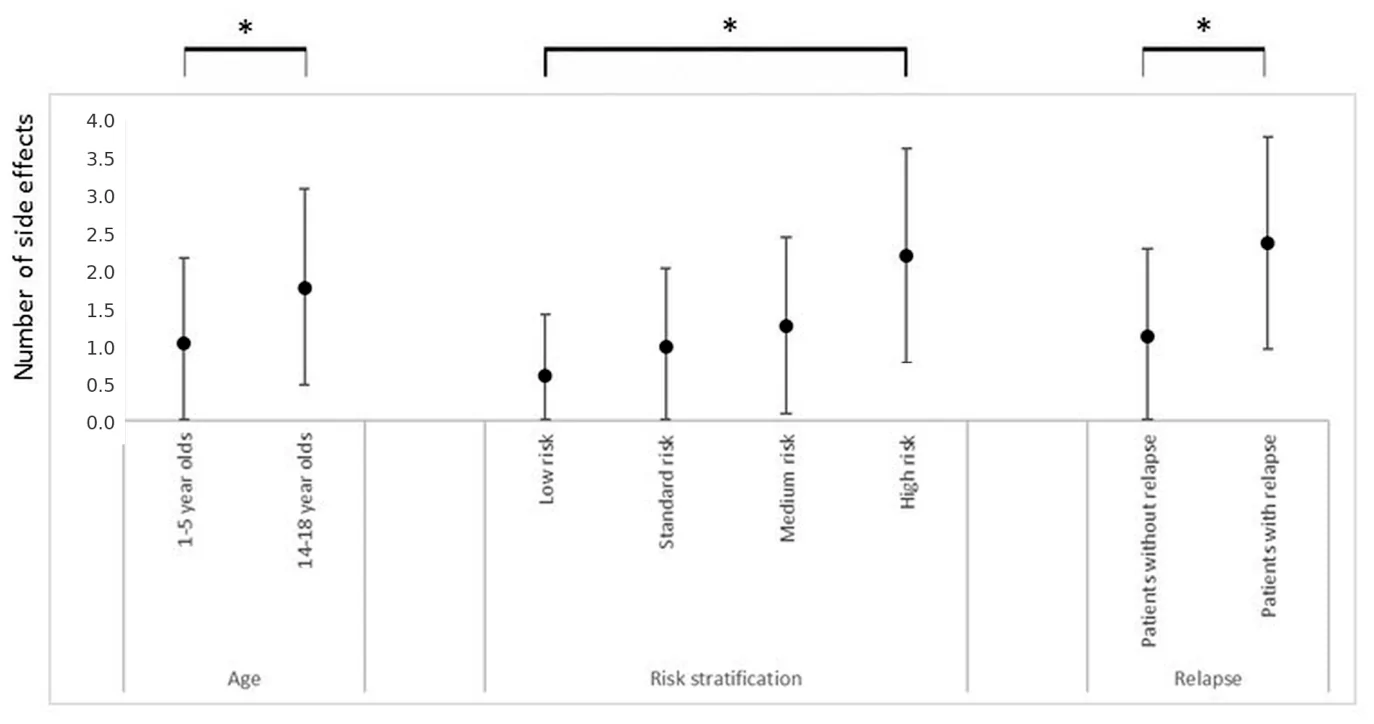
Analysis of risk factors for the occurrence of acute cardiovascular toxicities in ALL patients. Risk factors for cardiotoxicity were age at diagnosis >14 years, higher ALL risk score, and relapse of the oncologic disease. * *p* < 0.05.

**Table 1:** ALL: 69% (*n* = 58) showed at least one cardiotoxic side effect. Average of 1.26 (±1.23) side effects per patient. HL: 68% (*n* = 17) showed at least one cardiotoxic side effect. Average of 1.24 (±1.13) side effects per patient.

**ALL**						
**Number of side effects**	0	1	2	3	4	5
**Frequency**	31% (*n* = 26)	36.9% (*n* = 31)	15.5% (*n* = 13)	9.5% (*n* = 8)	6% (*n* = 5)	1.2% (*n* = 1)
**Hodgkin**						
**Number of side effects**	0	1	2	3		
**Frequency**	32% (*n* = 8)	32% (*n* = 8)	16% (*n* = 4)	20% (*n* = 5)		

## Data Availability

The original contributions presented in this study are included in the article. Further inquiries can be directed to the corresponding author.
